# Digital Twin Pathology of Skin Cancer

**DOI:** 10.1111/jocd.70576

**Published:** 2025-11-26

**Authors:** Clay J. Cockerell, Mohamad Goldust

**Affiliations:** ^1^ Department of Dermatology, Cockerell Dermatopathology Dallas Texas USA; ^2^ Department of Dermatology Yale University School of Medicine New Haven Connecticut USA

**Keywords:** digital twin, pathology, skin cancer

Dear Editor,

The field of dermatology is entering a transformative era where artificial intelligence (AI), computational modeling, and precision oncology converge to redefine how skin cancers are studied and managed. Among emerging innovations, digital twin pathology, a computational model of a patient's skin cancer, holds exceptional promise. By integrating clinical, dermoscopic, histopathological, and genomic data, this model continuously evolves with real‐world inputs, simulating disease behavior and therapeutic response in real time [1, 2].

Digital twins have already proven valuable in cardiology and intensive care for predictive monitoring. In dermatologic oncology, their relevance is amplified by the diversity of cutaneous malignancies such as melanoma, squamous cell carcinoma, and basal cell carcinoma. Using high‐resolution dermoscopy, whole‐slide histopathology, and next‐generation sequencing, AI‐driven algorithms synthesize a dynamic model of tumor biology. As new data are incorporated—follow‐up imaging, biopsies, or circulating biomarkers—the twin refines itself, providing a mirror of the tumor's evolving landscape [3, 4].

A defining feature of this approach is virtual treatment simulation. By modeling patient‐specific immune or genetic profiles, clinicians can predict responses to immunotherapy, targeted agents, or novel topical compounds before clinical exposure. For example, a digital twin of a melanoma can simulate how immune checkpoint inhibitors might enhance tumor clearance or anticipate when resistance mutations may arise, enabling early intervention and better outcomes.

Digital twin pathology can also enhance clinical decision support and research efficiency. Simulated patient cohorts can be used to refine trial design, test dosing regimens, and identify predictive biomarkers. Integration into electronic health records may allow dermatologists to conduct “what‐if” analyses during tumor board meetings, strengthening precision‐based care. Furthermore, digital twins could serve as powerful educational tools, offering dermatology trainees interactive insight into tumor dynamics and therapeutic responses (Table 1).

**TABLE 1:** Key components and applications of digital twin pathology in skin cancer.

Component	Description	Applications in skin cancer
Clinical and dermoscopic data	High‐resolution clinical photographs, dermoscopy, reflectance confocal microscopy	Early detection, lesion monitoring, digital phenotype characterization
Histopathology	Whole‐slide imaging of biopsies, AI‐assisted image analysis	Morphological replication, virtual re‐biopsy, tumor grading
Genomic and molecular data	Next‐generation sequencing, transcriptomics, proteomics	Modeling tumor evolution, predicting drug resistance, biomarker discovery
Patient‐specific factors	Immune profile, comorbidities, treatment history	Personalized therapy simulations, immunotherapy response prediction
Computational modeling	AI/ML algorithms integrating multimodal datasets	“What‐if” therapy simulations, prediction of relapse/progression
Clinical applications	Integration into EHR and tumor boards	Decision support, trial design, training and education

However, creating reliable twins requires large, diverse, and ethically sourced datasets. Bias, underrepresentation of skin of color, and privacy concerns remain major barriers. Transparent algorithm design, secure data governance, and clear regulatory frameworks are essential to ensure patient trust and clinical safety [5].

Digital twin pathology marks a paradigm shift from reactive treatment to proactive precision care in dermatologic oncology. By enabling virtual simulation and personalized prediction, this technology can minimize risk, optimize therapy, and transform how dermatologists approach cancer management. Dermatology, anchored in imaging, histopathology, and innovation is ideally positioned to lead this revolution toward truly individualized skin cancer care.

## Funding

The authors have nothing to report.

## Disclosure

We confirm that the manuscript has been read and approved by all the authors, that the requirements for authorship as stated earlier in this document have been met and that each author believes that the manuscript represents honest work.

## Ethics Statement

The authors have nothing to report.

## Consent

The authors have nothing to report.

## Conflicts of Interest

The authors declare no conflicts of interest.

## Data Availability

Data sharing not applicable to this article as no datasets were generated or analysed during the current study.
